# Effectiveness of Short Message Service Reminders on Timely Pick-up of Antiretroviral Therapy Among Consenting Persons with HIV in Zambézia Province, Mozambique

**DOI:** 10.1177/08892229251388415

**Published:** 2025-10-29

**Authors:** Erin Graves, Caroline De Schacht, Wu Gong, Sara Van Rompaey, Maria F.S. Alvim, Gaël Claquin, Bryan E. Shepherd, Ann F. Green, José A. Tique, Eurico José, Hélio Machabane, Eusébio Maposse, Magdalena Bravo, Anibal N. Fernando, C. William Wester

**Affiliations:** 1 Vanderbilt Institute for Global Health (VIGH), Vanderbilt University Medical Center (VUMC), Nashville, Tennessee, USA.; 2 Centro pela Saúde Global (C-Saúde), Maputo, Mozambique.; 3 Friends in Global Health (FGH), Maputo, Mozambique.; 4 Department of Biostatistics, Vanderbilt University Medical Center (VUMC), Nashville, Tennessee, USA.; 5 Zambézia Provincial Health Directorate (DPS-Z), Quelimane, Mozambique.; 6 Department of Medicine, Division of Infectious Diseases, Vanderbilt University Medical Center (VUMC), Nashville, Tennessee, USA.

**Keywords:** HIV, short message service, reminders, antiretroviral therapy, retention, Mozambique

## Abstract

A short message service (SMS) intervention was implemented in July 2016 at eight health facilities (HFs) within Zambézia Province, Mozambique. We assessed effectiveness by comparing antiretroviral therapy (ART) pick-up rates among persons with HIV (PWH) eligible for SMS reminders. All adult (≥15 years of age) PWH enrolled in ART services reporting cell phone access were offered SMS. PWH were sent messages 15, 7, and 2 days before scheduled ART pick-up appointments. Using routine data (July 2016–May 2018), mixed-effect logistic regression was used to determine adjusted odds ratios (aORs) of ART pick-up within 2, 6, and 59 days of scheduled appointments, adjusting for age, sex, adherence support group status, partner status, pregnancy, education, occupation, years on ART, HF, pick-up day, and year-week. Data were available for 18,941 scheduled ART pick-up clinic visits for 3,222 PWH (52% female) reporting cell phone access. Overall, 47% of men and 46% of women consented to receive SMS. PWH sent reminders had higher pick-up rates within 2 days of scheduled appointments compared with those not sent reminders (54% vs. 51%). Men sent reminders were more likely to pick-up within 2 days of scheduled appointments than men not sent reminders [aOR = 1.22 (1.09–1.37)]. No association was seen within 6 or 59 days of scheduled appointments [aOR = 1.09 (0.95–1.25), 0.90 (0.74–1.10)]. Women sent reminders had similar pick-up rates compared with women not sent reminders [aOR = 0.94 (0.85–1.05), 0.90 (0.79–1.03), 0.89 (0.72–1.10)]. In Zambézia Province, SMS reminders were associated with timely short-term ART pick-up rates among men, although this association attenuated with increased time from scheduled appointment. Study limitations included its observational (nonrandomized) nature and restricting analyses to individuals reporting phone access. SMS reminders may be an important approach for addressing the well-documented need for medication adherence support for males. Additional context-specific strategies are needed to ensure timely ART pick-up and improve retention.

## Introduction

As the ownership and use of mobile phones becomes more ubiquitous, health professionals begin to incorporate mobile phone technologies, such as short message service (SMS) reminders, for the provision of services.^1^ The mobile health (mHealth) movement has evolved to include interventions designed to improve patient follow-up and medication adherence, including consultation reminders and alerts of result availability, among other patient-centered uses.^1^ An increasing number of studies from resource-constrained settings, and fewer from low- and middle-income countries (LMICs), have been conducted to better understand the impact of mHealth interventions on patient retention, antiretroviral therapy (ART) adherence,^2^ and other essential human immunodeficiency virus (HIV) continuum of care outcomes.^3^

Among the various interventions aimed at improving adherence to ART, SMS reminders have been found effective at increasing adherence.^2–12^ Brief SMS messages sent on a scheduled basis as appointment reminders have been shown to improve participation in health consultations.^6,10,13–16^ A Botswana study found that a greater proportion (85%) of patients receiving SMS reminders had timely ART pick-ups from urban pharmacies compared with those not receiving reminders (70%), although the results were not statistically significant.^17^ SMS-based interventions for promoting engagement in health services are largely acceptable among patient populations,^4,18,19^ and studies indicate that SMS reminders are one of the more cost-effective options in LMICs when compared with calls and other reminder-based interventions.^2,16^

The Mozambique-based nongovernmental organization, Friends in Global Health (FGH), is a wholly owned subsidiary of the Vanderbilt University Medical Center (VUMC), providing technical support to the Ministry of Health (MOH) in the centrally located Zambézia Province. In 2016, an mHealth interventional study in Mozambique showed improved ART appointment attendance and retention in care with SMS cell phone messaging in urban areas in Maputo Province,^20^ and a 2017 study found patient and health care worker (HCW) acceptability of SMS text communication to promote HIV and tuberculosis treatment adherence also in the Maputo Province context.^21^ Informed by these findings, and equipped with newly developed systems, FGH piloted a novel intervention in Zambézia Province to provide automated SMS reminders to consenting eligible adult patients enrolled in HIV care with the goal of improving patients’ ART pick-up rates. Beginning in July 2016, an SMS reminder service was rolled out to FGH-supported health facilities (HFs) in Zambézia, with prioritization of HFs having large patient volumes for the phased implementation selected in collaboration with the Provincial Health Directorate of Zambézia (DPS-Z) authorities. We herein describe the subsequent evaluation, the primary objective of which was to assess the effectiveness of this SMS intervention by comparing ART pick-up rates among patients sent versus those not sent reminders at these locations.

## Methods

### Intervention design and implementation

Starting in July 2016, the automated SMS service began being rolled out to eight FGH-supported HFs in Zambézia Province. These included three district capitals (i.e., “headquarter”) HF sites of Namacurra, Mocubela, and Maganja da Costa districts, and five urban HFs in the provincial capital district, namely, Quelimane City: 17 de Setembro, 24 de Julho, 4 de Dezembro, Chabeco, and Coalane.

All persons with HIV (PWH) enrolled in ART services at these HFs who reported having access to a cell phone and who had provided permission to be contacted via text were eligible for and offered SMS reminders. Following standardized enrollment procedures, health counselors (HC) used a written script to explain the informed consent section of the MOH-approved form to ensure that patients were fully informed about the SMS service, including the timing and frequency of reminders and that the service was optional and they could withdraw from service enrollment at any time.

A module was created in Open Medical Records System (OpenMRS)™, the electronic patient database used to track patient’s HIV services, that identified eligible patients each morning at 9:00 AM, based on having a consent registered and the day of the next scheduled antiretroviral medication pick-up. Text reminders in Portuguese were automatically sent to identified eligible patients through a Global System for Mobile Communications (GSM) modem 15, 7, and 2 days prior to the scheduled ART pick-up visits. Sent messages read “[*First, Last Name*] has an appointment at the [*Health Facility*] on [*Day, Month, Year*],” with no further identifying information or reference to the purpose of appointment, to protect patients’ privacy/confidentiality. The content for the simple, uniform messages was reviewed and approved by technical team members with expertise in local context prior to implementation.

### Evaluation design and sampling strategy

An observational retrospective evaluation was done with the primary objective of assessing the association between the SMS reminder intervention and adult patients’ ART adherence, as measured by the timeliness of ART pickups. A secondary objective was to describe the uptake of SMS reminders among eligible adults.

Data were collected for PWH (≥15 years of age) enrolled in ART services between July 2016 (first month of SMS) and May 2018 in the eight HFs with SMS intervention (Fig. 1). To account for the potential selection bias related to cell phone ownership (i.e., those patients without, or without access to, a cell phone could not opt into the SMS service), we restricted the analysis to patients reporting access to a cell phone (i.e., they personally owned a cell phone or were able to consistently use a cell phone owned by someone else). Patients who were enrolled in ART services at these eight HFs but then transferred to receive ART services at a HF where the SMS intervention was not offered during the evaluation period were excluded from the analysis. Variables of interest included but were not limited to all scheduled and completed HF visit dates for ART pick-ups and consultations, as well as deidentified sociodemographic data.

**FIG. 1.**
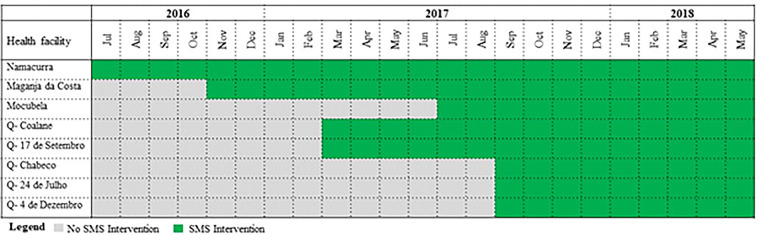
Short message service (SMS) intervention evaluation period (July 2016–May 2018), rural and urban health facilities.

The number of SMS messages sent were summarized per alert date, that is, the date of the scheduled ART pick-up being notified by the message. All available scheduled and actual ART pick-up dates from July 2016 to May 2018 were included as observations in the analysis to examine the likelihood of a timely ART pick-up based on the scheduled appointment date: within 2 days of scheduled appointment was considered *on-time*; within 6 days was considered *prior to defaulting*; and within 59 days was considered *prior to lost-to-follow-up* (LTFU) (per MOH definitions).^22^

### Data collection and management

Data used were routinely collected by district and provincial teams for programmatic monitoring and reporting purposes. All data were subject to routine verification processes conducted by trained members of FGH’s Monitoring and Evaluation (M&E) team. Data were stored securely on password-protected databases and servers at FGH offices, and could only be accessed by FGH M&E and Health Information System teams. Deidentified data were extracted from the existing OpenMRS electronic medical record databases into a restricted dataset by qualified FGH employees and securely sent to VUMC statisticians for analysis.

### Statistical analyses

A mixed-effects logistic regression model was applied to ART pick-up outcomes within 2, 6, and 59 days of scheduled appointments to evaluate the effect of SMS intervention on timely ART pick-up. Models were restricted to pick-ups by patients reporting access to a cell phone. Unadjusted and adjusted odds ratios (aORs) were reported from the models. Fixed-effect covariates included SMS service (time-varying binary variable), sex, age, community adherence support group [CASG] status, partner status, pregnancy, education, occupation, years on ART, weekday of appointment, and HF. An interaction term between sex and SMS service was also included, given the behavior differences between females and males related to ART pick-up and cell phone ownership in Mozambique, where females have been found to perform better in adhering to ART^23,24^ and a higher proportion of males than females have cell phones.^25^ Random effects included the patient identification number to account for the similar behavior of multiple ART pick-ups by individual patients, and the year-week of pick-up (e.g., 2016—week 1, 2016—week 2,…) to account for temporal influence on the pick-up pattern in a parsimonious manner. Reported *p* values and 95% confidence intervals (CI) are not adjusted for multiple comparisons. As not all patients reporting phone access consented to receive the SMS service, a sensitivity analysis was performed on the population of consented patients. The analysis was performed using R statistical software and the “lme4” package.^26,27^

### Ethical considerations

The evaluation was conducted according to national and international research ethics norms to ensure that the rights of every patient were respected. Prior to data collection, a blanket protocol for evaluating program performance through secondary data analyses received ethics approvals from the Institutional Health Bioethics Committee of Zambézia (CIBS-Z), Mozambique (Ref: 02/CIBS-Z/16), the VUMC Institutional Review Board (#170970), and the funding agency (Centers for Disease Control and Prevention [CDC]).

## Results

### Descriptive results and SMS service uptake

We used 18,941 eligible scheduled ART pick-ups from 3,222 patients who met the inclusion criteria (i.e., ≥15 years of age, from one of eight selected HFs, reported phone access, between July 2016 and May 2018). Among all eligible patients, 1,664 (51.6%) were female, and 1,498 (46.5%) opted to enroll in the SMS service (Table 1). Rates of SMS enrollment were similar between eligible males (47.2%) and females (45.9%). Initiation of SMS programs was staggered across sites (Fig. 1). Overall, SMS reminders were sent for 6,385 (34%) of the scheduled ART pick-ups and were not sent for the remaining 12,556 (66%).

**Table 1.** Descriptive Sociodemographic and Clinical Patient Characteristics Using Univariate Analysis of Short Message Service (SMS) Uptake Among All Eligible Patients, by Service Uptake (Zambézia Province, Mozambique, July 2016–May 2018)

Variable	Declined SMS	Accepted SMS	*p* Value
Number of patients			
*N* (%)	1,724 (53.5%)^a^	1,498 (46.5%)	
Sex			
Male	823 (47.7%)	735 (49.1%)	.474
Female	901 (52.3%)	763 (50.9%)	
Age at ART pick-up (median [IQR])	30.2 [24.8, 37.0]^b^	30.8 [25.3, 38.1]	.008
Age at scheduled ART pick-up date (%)			
15–24 years	441 (25.6%)	343 (22.9%)	.163
25–49 years	1,167 (67.7%)	1,041 (69.5%)	
≥50 years	116 (6.7%)	114 (7.6%)	
Partner status			
Negative	1,599 (92.7%)	1,362 (90.9%)	.067
Positive	125 (7.3%)	136 (9.1%)	
Pregnancy status (all patients)			
No	1,461 (84.7%)	1,311 (87.5%)	.027
Yes	263 (15.3%)	187 (12.5%)	
Years on ART			
<6 months	1,678 (97.3%)	1,491 (99.5%)	<.001
<1 year	38 (2.2%)	6 (0.4%)	
≥1 year	8 (0.5%)	1 (0.1%)	
Education level			
None	127 (7.4%)	128 (8.5%)	.026
Primary	592 (34.3%)	456 (30.4%)	
Secondary	889 (51.6%)	831 (55.5%)	
Missing^c^	116 (6.7%)	83 (5.5%)	
Occupation			
Employed	731 (42.4%)	665 (44.4%)	.137
Homemaker	541 (31.4%)	451 (30.1%)	
Farmer	190 (11.0%)	160 (10.7%)	
Student	175 (10.2%)	173 (11.5%)	
Unemployed	5 (0.3%)	5 (0.3%)	
Retired	1 (0.1%)	1 (0.1%)	
Missing	81 (4.7%)	43 (2.9%)	
HF			
Maganja da Costa sede	205 (11.9%)	96 (6.4%)	<.001
Mocubela sede	33 (1.9%)	83 (5.5%)	
Namacurra sede	250 (14.5%)	215 (14.4%)	
17 de Setembro^d^	306 (17.7%)	249 (16.6%)	
24 de Julho^d^	269 (15.6%)	151 (10.1%)	
4 de Dezembro^d^	295 (17.1%)	263 (17.6%)	
Chabeco^d^	184 (10.7%)	144 (9.6%)	
Coalane^d^	182 (10.6%)	297 (19.8%)	

a For categorical variables, frequency (percentage) is shown.

b For continuous variables, median (IQR) is shown.

c Missing category appears only when there are missing values for a variable.

d Denotes HF sites in Quelimane City district, urban provincial capital (vs. rural district headquarters HF).

ART, antiretroviral therapy; HF, Health facility; IQR, interquartile range; SMS, short message service.

Table 1 shows descriptive characteristics of the evaluation population, by service uptake. Patients accepting SMS reminders were 50.9% female, compared with patients who declined (52.3% female). Among patients accepting, 22.9% were between 15 and 24 years, 69.5% were between 25 and 49 years, and 7.6% were ≥50. Nearly 56% of patients who accepted SMS completed some or all of secondary school, compared with 52% of those declining. Approximately 9% of patients accepting SMS reminders reported having a partner who was also seropositive, compared with 7.3% of patients who declined SMS. A slightly greater proportion of female patients who declined SMS reminders reported being pregnant (15.3%) than did those who accepted SMS (12.5%). Most patients had been on ART for less than 6 months, regardless of SMS uptake (99.5% and 97.3%, respectively). SMS acceptance ranged from 32% at the Maganja da Costa district capital HF to 72% at the Mocubela district capital HF.

With regard to ART pick-up characteristics (Table 2), the proportion of pick-ups that occurred within 2 days of the scheduled date was greater for patients sent SMS (54.1%) compared with patients who were not sent (50.8%). Greater proportions of ART pick-ups took place when an SMS reminder was sent for a scheduled visit day that fell on a Wednesday, Thursday, Friday, or Saturday (16%, 20%, 25%, and 6%, respectively) compared with pick-ups scheduled on these days without a reminder (15%, 19%, 21%, and 5%, respectively). For ART pick-ups by pregnant females, the proportion for which SMS was not sent (16%) was slightly higher than the proportion for which SMS was sent (14%).

**Table 2.** Descriptive Sociodemographic and Clinical Patient Characteristics Using Univariate Analysis of All Scheduled Antiretroviral Therapy Pick-ups Included in the Analysis, by Status of Pick-up Reminder Sent (Zambézia Province, Mozambique, July 2016–May 2018)

Variable	Level	Scheduled ART pick-ups		
		SMS reminder was not sent	SMS reminder was sent	*p* Value
Number of ART pick-ups	*N*	12,556	6,385	
Sex	Male	5,607 (47.7%)^a^	2,959 (46.3%)	.029
	Female	6,949 (55.3%)	3,426 (53.7%)	
ART pick-up pattern^b^	≤2 days	6,379 (50.8%)	3,453 (54.1%)	<.001
	3–6 days	3,347 (26.7%)	1,606 (25.2%)	
	7–59 days	1,817 (14.5%)	827 (13.0%)	
	≥60 days or never	1,013 (8.1%)	499 (7.8%)	
Age at ART pick-up	Years (median [IQR])	30.7 [25.3, 37.7]^c^	31.6 [26.3, 39.0]	<.001
Age at scheduled ART pick-up date	15–24 years of age	2,956 (23.5%)	1,293 (20.3%)	<.001
	25–49 years of age	8,596 (68.5%)	4,501 (70.5%)	
	≥50 years of age	1,004 (8.0%)	591 (9.3%)	
Enrolled in CASG	No	11,605 (92.4%)	5,978 (93.6%)	.003
	Yes	951 (7.6%)	407 (6.4%)	
Partner status	Negative	11,596 (92.4%)	5,862 (91.8%)	.196
	Positive	960 (7.6%)	523 (8.2%)	
Pregnancy status (all patients)	No	10,522 (83.8%)	5,499 (86.1%)	<.001
	Yes	2,034 (16.2%)	886 (13.9%)	
ART duration	<6 months	7,779 (62.0%)	3,427 (53.7%)	<.001
	<1 year	3,105 (24.7%)	2,235 (35.0%)	
	≥1 year	1,672 (13.3%)	723 (11.3%)	
ART duration	Years (median [IQR])	0.3 [0.2, 0.7]	0.5 [0.2, 0.7]	<.001
Health facility (HF)	Maganja da Costa	1,759 (14.0%)	500 (7.8%)	<.001
	Mocubela	559 (4.5%)	446 (7.0%)	
	Namacurra	2,115 (16.8%)	809 (12.7%)	
	17 de Setembro^d^	1,753 (14.0%)	1,065 (16.7%)	
	24 de Julho^d^	1,542 (12.3%)	639 (10.0%)	
	4 de Dezembro^d^	2,254 (18.0%)	1,119 (17.5%)	
	Chabeco^d^	1,132 (9.0%)	539 (8.4%)	
	Coalane^d^	1,442 (11.5%)	1,268 (19.9%)	
Weekday of scheduled ART pick-up	Monday	2,772 (22.1%)	1,051 (16.5%)	
	Tuesday	1,462 (11.6%)	622 (9.7%)	
	Wednesday	1,839 (14.6%)	1,038 (16.3%)	
	Thursday	2,413 (19.2%)	1,291 (20.2%)	
	Friday	2,657 (21.2%)	1,571 (24.6%)	
	Saturday	634 (5.0%)	410 (6.4%)	
	Sunday	779 (6.2%)	402 (6.3%)	<.001

a For categorical variables, frequency (percentage) is shown.

b Defined as the number of days from the scheduled ART pick-up date.

c For continuous variables, median (IQR) is shown.

d Denotes HF sites in Quelimane City district, urban provincial capital (versus rural district headquarters HF).

CASG, community adherence support group; ART, antiretroviral therapy; HF, health facility; IQR, interquartile range; SMS, short message service.

Table 3 and Figure 2 show aORs for ART pick-up derived from a mixed-effects logistic regression model. Overall, the odds of timely pick-up were fairly similar between those sent SMS reminders and those not sent SMS reminders in multivariable analyses. Overall, the only significant association between SMS and ART pick-up was seen for males, whereby males sent SMS reminders had 22% [95% CI 9%–37%] higher odds of picking up ART within 2 days of scheduled appointments than males not sent SMS reminders. For pick-up within 6 days and 59 days of the scheduled date, there was little evidence of an association with SMS reminders for males (aOR = 1.09 [0.95–1.25]; aOR = 0.90 [0.74–1.10], respectively). No SMS reminder effect was seen on the timeliness of ART pick-up among females (ART pick-up within 2 days: aOR = 0.94 [0.85–1.05]; within 6 days: aOR = 0.9 [0.79–1.03]; within 59 days: aOR = 0.89 [0.72–1.10].

**FIG. 2.**
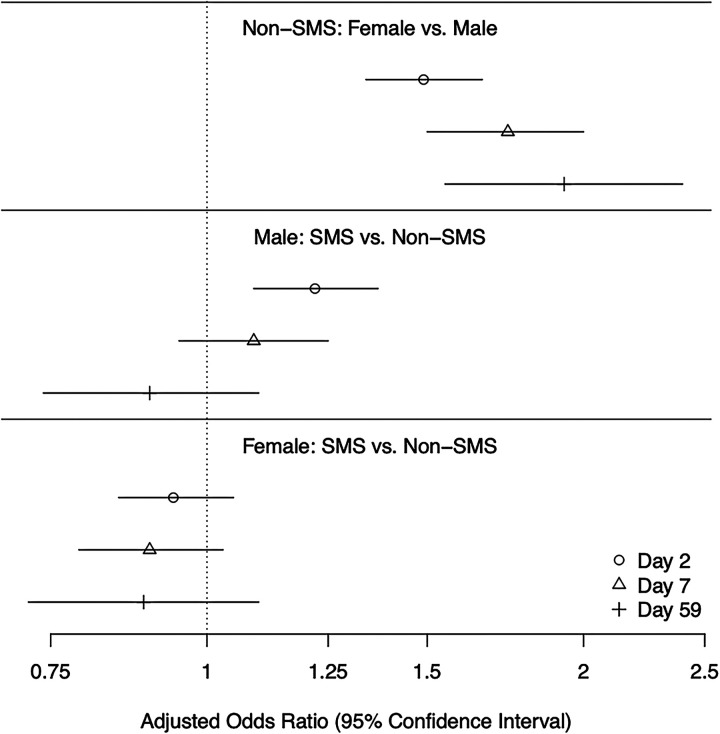
Forest plot for confidence intervals (CIs) of adjusted odds ratios (aORs), by sex and short message service (SMS) effect, for antiretroviral therapy (ART) pick-ups within 2, 6, and 59 days of scheduled appointment. The x-axis is on a logarithmic scale.

**Table 3.** Results for Adjusted Odds Ratios for Antiretroviral Therapy Pick-up from Mixed-Effects Logistic Regression Model, by 2, 6, and 59 Days from Scheduled Appointment Date (Zambézia Province, Mozambique; July 2016–May 2018)

Factor	ART pick-up within 0–2 days of scheduled appointment		ART pick-up within 6 days of scheduled appointment		ART pick-up within 59 days of scheduled appointment	
	Adjusted OR^a^ [95% CI]	*p* Value	Adjusted OR^a^ [95% CI]	*p* Value	Adjusted OR^a^ [95% CI]	*p* Value
SMS and sex^b^						
Females without SMS vs. males without SMS	1.49 [1.34–1.66]	<0.001	1.74 [1.50–2.00]	<0.001	1.93 [1.55–2.40]	<.001
Males with SMS vs. males without SMS	1.22 [1.09–1.37]	<0.001	1.09 [0.95–1.25]	0.2	0.90 [0.74–1.10]	.29
Females with SMS vs. females without SMS	0.94 [0.85–1.05]	0.28	0.90 [0.79–1.03]	0.12	0.89 [0.72–1.10]	.28
Age						
15–24 years	REF		REF		REF	
25–49 years	1.15 [1.04–0.28]	0.01	1.28 [1.11–1.47]	<0.001	1.72 [1.40–2.12]	<.001
50+ years	1.43 [1.20–1.71]	<0.001	1.75 [1.38–2.22]	<0.001	2.61 [1.82–3.75]	<.001
Enrolled in CASG						
No	REF		REF		REF	
Yes	1.76 [1.46, 2.12]	<0.001	2.24 [1.73, 2.91]	<0.001	9.09 [5.50, 15.03]	<.001
Partner HIV status						
Negative	REF		REF		REF	
Positive	0.97 [0.84, 1.13]	0.72	1.20 [0.98, 1.47]	0.07	1.51 [1.10, 2.08]	.01
Pregnancy status						
No	REF		REF		REF	
Yes	0.89 [0.79, 1.01]	0.07	0.95 [0.81, 1.13	0.56	1.50 [1.15, 1.95]	<.01
Education level completed						
None	REF		REF		REF	
Primary	1.08 [0.92, 1.26]	0.37	1.03 [0.83, 1.27]	0.8	1.07 [0.77, 1.47]	.69
Secondary	1.15 [0.98, 1.35]	0.08	1.17 [0.94, 1.45]	0.15	1.26 [0.91, 1.73]	.16
ART duration						
<6 months	REF		REF		REF	
6–12 months	0.93 [0.86, 1.01]	0.09	0.88 [0.80, 0.97]	0.01	0.85 [0.73, 0.99]	.04
>12 months	0.85 [0.75, 0.96]	0.01	0.84 [0.73, 0.97]	0.02	0.73 [0.59, 0.92]	.01

a Variables adjusted for included sex, age, CASG status, partner status, pregnancy, education, occupation, years on ART, weekday of appointment, HF.

b An interaction between short message service (SMS) and sex was included in the model; these contrasts are derived from the fitted model. (For all other factors, no interaction was found with SMS and was thus not included in the model and results are reported as overall.)

ART, antiretroviral therapy; CASG, community adherence support group; HF, health facility; IQR, interquartile range; SMS, short message service.

In general, females were more likely to pick up ART in a timely manner than males. Among patients not receiving SMS reminders, females were significantly more likely than males to pick up their ART within 2 (aOR = 1.49 [1.34–1.66], *p* < .001), 6 (aOR = 1.74 [1.50–2.00], *p* < .001), and 59 days (aOR = 1.93 [1.55–2.40], *p* < .001) of scheduled appointments. Among those sent SMS reminders, approximately 80% of females had picked up their ART by 5 days after their scheduled appointment, compared with approximately 8 days for 80% of males to pick up their ART. Females who were pregnant were significantly more likely to pick up their ART within 59 days of their scheduled appointment compared with nonpregnant females (aOR = 1.50 [1.15–1.95], *p* < .01). This positive association for timeliness of ART pickup was not seen, however, for pregnant females’ pick-ups within 2 or 6 days of scheduled appointments (aOR = 0.89 [0.79–1.01], *p* = .07, and 0.95 [0.81–1.13]; *p* = .56, respectively).

Overall (i.e., regardless of SMS status), compared with younger adult patients (15–24 years of age), adults 25–49 years of age were more likely to pick up their ART within 2 (aOR = 1.15 [1.04–1.28], *p* < .01), 6 (aOR = 1.28 [1.11–1.47], *p* < .001), and 59 days (aOR = 1.72 [1.40–2.12], *p* < .001) of scheduled appointments. Older adults (≥50 years of age) had the greatest likelihood of ART pick-up within 2 (aOR = 1.43 [1.20–1.71], *p* < .001), 6 (aOR = 1.75 [1.38–2.22], *p* < .001), and 59 days of scheduled appointments (aOR = 2.61 [1.82–3.75], *p* < .001) compared with younger adults (15–24 years of age).

Patients receiving ART via CASG were significantly more likely to pick up their ART within 2 (aOR = 1.76 [1.46–2.12], *p* < .001), 6 (aOR = 2.24 [1.73–2.91], *p* < .001), and 59 days (aOR = 9.09 [5.50–15.03], *p* < .001) of their scheduled appointments compared with patients not in a CASG. Patients who reported having a seropositive partner were significantly more likely to pick up ART within 59 days of their scheduled appointment (aOR = 1.51 [1.10–2.08], *p* = .01) compared with patients who reported having a seronegative partner. Patients who had been on ART for more than 12 months were significantly less likely to pick up their ART within 2 (aOR = 0.85 [0.75–0.96], *p* = .01), 6 (aOR = 0.84 [0.73–0.97], *p* = .02), and 59 days (aOR = 0.73 [0.59–0.92], *p* = .01) of their scheduled appointments compared with patients who had been enrolled in ART services for less than 6 months.

## Discussion

This evaluation aimed to assess the effectiveness of an SMS reminder intervention via comparison of ART pick-up rates among patients sent versus those not sent SMS in Zambézia Province. Overall, we found that the proportion of pick-ups occurring within 2 days of the scheduled date was slightly higher when an SMS reminder was sent compared to when not sent (54% vs. 51%). Our findings indicate that the SMS intervention appears to have a short-term positive effect on improving the timeliness of ART pick-ups by day 2 after the scheduled appointment among males sent SMS, however, by day 6 and day 59 after the scheduled appointment, the effect was no longer observed in males sent SMS. No SMS effect was seen for ART pick-ups by females.

There is a well-recognized concern that males in this region have lower rates of ART adherence and retention in care, especially adolescent/younger males. A 2017 program evaluation analyzing HIV care continuum outcomes among adolescents and young adults in Zambézia found that males were less likely to adhere to ART pick-ups, and younger males in particular need additional support to improve medication adherence and retention in care rates, and reduce their mortality risk.^23^

Overall, patients who were either female, older aged, or enrolled in CASG were more likely to have on-time ART pick-ups, not default, and not be LTFU compared with male, younger aged, and non-CASG patients, respectively. Women had higher odds for picking up ART regardless of SMS, and no effect was found when SMS reminders were sent to females. This finding can be interpreted in a variety of ways related to sociocultural factors that may have been at play: Females in Zambézia may be less likely to have consistent phone access and when they do have access it may not be their phone but rather their spouse’s or another family member’s, such that sent reminder messages may have been received by their spouse or another person and not relayed appropriately to the intended female recipient; females might have less need for reminders as they perform better at timely ART pick-ups; and females may attend the HF more frequently (than their male counterparts), facilitating greater convenience for retrieving ART. Moreover, if females in this area have high ART adherence rates overall, there may be a ceiling effect that limits the ability to measure any additional improvements in this subpopulation. Pregnancy may be a protective factor for obtaining ART prior to defaulting in HIV care; however, status as pregnant may not play a role for SMS sent within the short time interval (i.e., within 2–6 days) of scheduled pick-up date. Despite a lack of evidence of significant improvements from SMS for HIV outcomes among perinatal women living with HIV, other advantages found for this group, such as improved time to postpartum contraception uptake,^28,29^ need to be further investigated.

Overall, our results show that a considerable proportion of eligible patients are inclined to accept SMS in Zambézia, with slightly less than half of all eligible adult patients accepting these services, and males slightly more likely to accept than females. Further investigation is necessary to identify facilitating factors for SMS uptake, especially among males and young adults, and particularly if any social or cultural differences such as perceived stigma or privacy concerns may serve as barriers to SMS uptake.

There is a considerable need to explore the effects of sending patient-specific, tailored SMS messages to patients in high-risk groups (e.g., pregnant and lactating women [PLW], male partners, infants exposed to HIV [HEI], youth) to support favorable HIV continuum-of-care outcomes.^2,6,7,14–16,30–33^ A meta-analysis of controlled trials showed that interventions involving personalized messages had greater effects on adherence when compared with uniform or generalized message content.^7^ SMS reminders aimed at PLW and their male partners, with tailored messages promoting prevention of mother-to-child transmission care uptake should be piloted to assess efficacy at improving maternal and infant outcomes and increasing male partner engagement in this critical window.^32–36^ Inclusion of SMS reminders in alternative adherence support strategies and multicomponent interventions needs to be considered to promote adherence and retention; a systematic review found that interventions offering SMS reminders and promotional health information are more effective at reducing missed HF appointments than only reminders,^15^ and a recent study in Mozambique showed that patients receiving a four-pronged intervention, including SMS reminders, were more likely to be retained in care after 1 year.^37^ Further research exploring the cost-effectiveness of SMS reminders—as a single-pronged intervention and within multipronged interventions aimed at improving patient retention—is warranted in this majoritively rural and resource-limited central region of Mozambique.

Implementation and/or modification of interventions effective in promoting ART adherence that also maintain compliance with prevention and mitigation strategies for emergency pandemics such as the coronavirus disease of 2019 (COVID-19) will be critical.^38^ To reduce COVID-19 spread among patients and HCWs, the MOH provided guidance for the rapid expansion of 3-monthly ART dispensation to decrease the frequency of HF visits. With this alteration to HIV follow-up and appointment frequency, provision of SMS reminders to promote timely ART pick-up is more important than ever, particularly for males and others most at-risk for attrition.

We acknowledge several limitations for this evaluation. First, the programmatic data used in this analysis could only inform if SMS messages were “sent”; we were unfortunately unable to assess if the messages were read, or by whom. In addition, to account for the selection bias due to phone ownership/access, the analysis was restricted to only patients reporting phone access. This conservative approach was adopted to achieve a more appropriate and representative comparison in the absence of utilizing matching comparisons. However, this limited our sample size for ART pick-ups and our ability to understand the effect SMS reminders could potentially have on the general population, as many in this region do not have access to a phone, perhaps disproportionately females, those with less economic resources, or those with less formal education.

Although increasing, mobile coverage/access in LMICs remains low compared with high-income countries: by 2017, per 100 inhabitants, there were 127 mobile-cellular telephone subscriptions in resource-replete settings, compared with 99 in resource-constrained settings, and only 69 in the most severely resource-constrained settings.^39^ Country-specific data from 2017 estimated that Mozambique had a mobile-cellular subscription rate of nearly 40 per 100 inhabitants,^40^ with an estimated 37% of males and 26% of females owning a mobile-cellular telephone.^25^ As access to mobile technologies is a key predeterminant to studying the effects of mobile/SMS interventions, further evaluation (including qualitative inquiries) is needed to investigate differences for those with and without cell phone ownership, and factors contributing to differences.

An additional selection bias existed related to the voluntary nature of accepting SMS reminders in this observational study. Due to the lack of randomization, it is possible that associations seen with picking up ART were related to other factors linked to patient-level differences. In addition, associations between confounder variables and ART pick-up may also not be causal.^41^ Since only 47% of eligible patients consented to SMS in this evaluation period, it should be explored whether access to a cell phone is a barrier issue or if some patients prefer not to receive messages for specific reasons. Conversely, it would be ideal to account for differences in outcomes among those who owned the phone versus those who reported access to a phone they did not own, as studies have highlighted the importance of addressing contextual factors of phone sharing and confidential disclosures.^35^

Lastly, the evaluation included a limited intervention period at selected sites; as such, we are unable to draw conclusions or make inferences regarding patients’ long-term behavior or outcomes such as becoming LTFU, and we recognize the importance of longer evaluation periods to assess the efficacy of SMS interventions on patient outcomes in this context.^3^

## Conclusions

In Zambézia Province, Mozambique, SMS reminders provided a significant short-term positive effect on timely ART pick-up among accepting males until 2 days after the scheduled appointment, although this effect diminished with increased time from the scheduled pick-up date. Overall, females had a higher likelihood for ART pick-up at each time interval, however, no additive effect was seen with SMS reminders. Older age also had a consistent positive effect on timely ART pick-up among all adults, regardless of SMS reminders.

Our findings support efforts to focus on SMS program adaptation to target males and youths, as well as program expansion via patient-centered messaging for high-risk groups, including PLW, HEI, and patients defaulting from care, as such messaging has been shown to be associated with increased retention in care.^6,15,21,42^ We plan to continue efforts to inform regional public health officials and policymakers of these contextual findings for consideration of adopting a similar and/or expanded SMS strategy to improve patient adherence. The proposed content for such reminder and reengagement strategies should be developed in collaboration with local experts and patient advocates to ensure that effective messaging is tailored to these key patient populations.^34^

## Authors’ Contributions

E.G.: Conceptualization (supporting), writing—original draft (lead), and writing—review and editing (equal). C.D.S.: Conceptualization (supporting), writing—original draft (supporting), and writing—review and editing (equal). W.G.: Conceptualization (supporting), formal analysis (lead), methodology (lead), visualization (lead), and writing—review and editing (equal). S.V.R.: Conceptualization (supporting), writing—original draft (supporting), and writing—review and editing (equal). M.F.S.A.: Conceptualization (supporting), data curation (supporting), and project administration (supporting). G.C.: Conceptualization (supporting), supervision (lead), and writing—review and editing (equal). B.E.S.: Conceptualization (supporting), formal analysis (supporting), methodology (supporting), and writing—review and editing (equal). A.F.G.: Conceptualization (supporting) and writing—review and editing (equal). J.A.T.: Supervision (supporting) and writing—review and editing (equal). E.J.: Data curation (supporting), project administration (lead), and software (lead). H.M.: Data curation (supporting), project administration (supporting), and software (supporting). E.M.: Data curation (lead) and project administration (supporting). M.B.: Conceptualization (supporting). A.N.F.: Conceptualization (supporting) and writing—review and editing (equal). C.W.W.: Conceptualization (lead), writing—original draft (supporting), and writing—review and editing (equal).

## Data Availability

The data that support the findings of this evaluation are owned by the Mozambique MOH. Restrictions apply to the availability of these data, which were used under authorization by the Institutional Health Bioethics Committee of Zambézia (Comité Institucional de Bioética para a Saúde-Zambézia, or CIBS-Z) and the MOH for this evaluation. The data are not publicly available as the MOH has not given its permission for authors to share the data. Data requests can be made by contacting the National Committee for Health Bioethics (Comité Nacional de Bioética para a Saúde, or CNBS): Ministry of Health, 2nd floor; Eduardo Mondlane Ave./Salvador Allende Ave. Maputo, Mozambique; cnbs.mocambique@gmail.com
